# Effects of Ozone Therapy on Adverse Events and Inflammatory Markers in Patients With Hepatocellular Carcinoma Undergoing Interventional Therapy

**DOI:** 10.1155/grp/9953993

**Published:** 2025-11-12

**Authors:** Minghui Zhu, Yujie Ran, Yufei Huang, Huan Zhao, Yuan Chao, Rong Fan, Xiaowei Chen

**Affiliations:** Department of Infectious Diseases, Nanfang Hospital, Southern Medical University, Guangzhou, China

**Keywords:** adverse events, hepatocellular carcinoma, interventional therapy, ozone therapy

## Abstract

**Background:**

There has been very limited investigation regarding reduced interventional therapy–related adverse events in patients with hepatocellular carcinoma (HCC). This study was aimed at evaluating the effect of ozone therapy as an adjunctive treatment on inflammation markers and adverse events in patients with HCC receiving interventional therapy.

**Methods:**

Three hundred and forty-two patients with HCC undergoing interventional therapy were enrolled from December 2020 to June 2023, of which 221 patients received rectal ozone insufflation therapy (ozone cohort), and the other 121 patients did not receive ozone therapy (control cohort). The information on treatment-related adverse events (TRAEs) was retrieved and analyzed.

**Results:**

In the study, most clinical characteristics between the ozone and control cohorts showed no significant differences. In the ozone cohort, 122 patients (55.2%) reported TRAEs of any grade, compared with 67 (55.4%) patients in the control cohort (*p* > 0.05). In terms of specific TRAE incidence, no distinctiveness was found in incidence of other TRAEs. Furthermore, multivariate logistic regression revealed that higher levels of AFP (OR, 1.82; 95% CI, 1.13–2.94; *p* = 0.014) and ALT/AST ratio (OR, 2.02; 95% CI, 1.04–3.91; *p* = 0.037) were independently correlated with increased risk of total TRAEs. Based on similar levels of laboratory parameters with patients with HCC before treatment, there were no significant differences in these biomarker levels posttreatment between the ozone and control cohorts.

**Conclusion:**

Ozone therapy did not significantly decrease the incidence of adverse events or mitigate the increase in inflammatory markers. Further research with a larger sample size is warranted.

## 1. Introduction

Primary liver cancer ranks as the sixth most commonly diagnosed cancer and the third leading cause of cancer-related deaths worldwide, accounting for over 900,000 new cases and approximately 830,000 deaths in 2020 [1]. Hepatocellular carcinoma (HCC), the most prevalent form of primary liver cancer, is also among the top three causes of cancer-related mortality in most countries [2]. Unfortunately, a significant proportion of patients with HCC are diagnosed at advanced stages, often when the disease is unresectable [3]. Transarterial chemoembolization (TACE) and hepatic artery infusion chemotherapy (HAIC) are widely used and effective treatments for unresectable HCC [4, 5], owing to their satisfactory efficacy and acceptable tolerability, which support their long-term application. However, these interventional treatments are often associated with side effects such as pain, fever, and vomiting during or after the procedure. While these adverse effects can generally be alleviated with supportive care and medication, they nonetheless impact the quality of life for patients with HCC. To prolong survival, considerable efforts have been directed toward improving treatment methods and enhancing therapeutic efficacy. However, relatively less attention has been given to minimizing unnecessary adverse events in patients with HCC undergoing interventional treatments [6]. Therefore, enhancing the safety profile of these therapies remains a critical area for further improvement.

Ozone is an allotropic form of oxygen, consisting of three oxygen atoms, and has been widely utilized in various medical applications, including the treatment of musculoskeletal disorders, pain management, and cancer. For example, a study demonstrated that adjuvant ozone therapy significantly improved symptoms in a high percentage of cancer survivors experiencing persistent or refractory side effects induced by radiotherapy and chemotherapy. Furthermore, ozone treatment was associated with a notable reduction in toxicity grade among these patients [7]. Ozone therapy also has applications in the field of liver disease. In a study involving 223 patients with chronic hepatitis C, it was found that after 12 weeks of ozone treatment, both the fibrosis stage and inflammation grade significantly decreased [8]. However, despite these encouraging results, the outcomes of studies on ozone therapy remain inconclusive and controversial. For instance, one study reported that ozone therapy had a detrimental effect on liver damage induced by iron, as evidenced by increased apoptosis in histopathological analysis compared with the control group [9]. Consequently, whether ozone therapy can effectively reduce the incidence of treatment-related adverse effects remains uncertain and warrants further investigation.

In this study, we aimed to evaluate the effects of ozone therapy as an adjunctive treatment in reducing adverse events and modulating inflammation markers in patients with HCC undergoing interventional therapy.

## 2. Patients and Methods

### 2.1. Study Population

**Figure 1:**
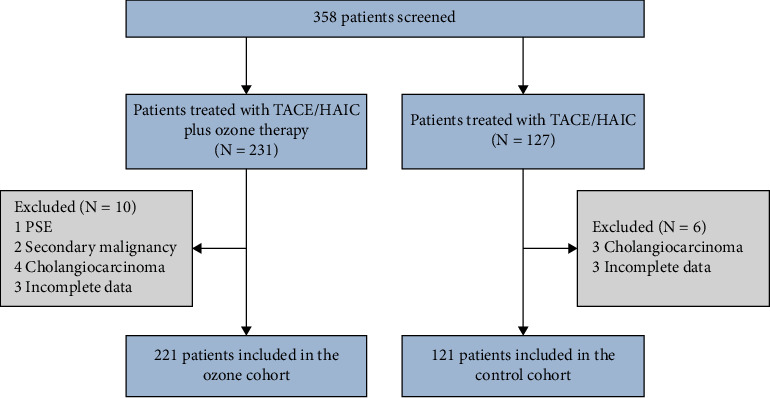
Flow of patients included in the analysis. TACE, transcatheter arterial chemoembolization; HAIC, hepatic artery infusion chemotherapy; PSE, partial of splenic embolization.

Between December 2020 and June 2023, a total of 342 patients with HCC undergoing interventional therapy were enrolled from the Liver Tumor Center, Department of Infectious Diseases, Nanfang Hospital, Southern Medical University, Guangzhou, China. Patients were eligible if they were over 18 years, diagnosed with HCC, treated with TACE or HAIC, and willing to provide personal information. Exclusion criteria included patients with other malignant diseases or solid tumors. Participants were then divided into two cohorts based on their treatment method: the ozone cohort and the control cohort (Figure 1). The ozone cohort received ozone therapy in addition to the treatment provided to the control cohort.

The study was approved by the Medical Ethics Committee of Nanfang Hospital, Southern Medical University. All patients provided written informed consent to participate.

### 2.2. Data Collection

Clinical data collection included demographic factors (age, sex, and body mass index [BMI]), Child–Pugh class, Barcelona Clinic Liver Cancer (BCLC) stage, Eastern Cooperative Oncology Group (ECOG) performance status, and the presence of cirrhosis.

Serological inflammatory markers and the biochemical parameters, including white blood cell (WBC) count, neutrophil (NEU) count, platelet (PLT) count, alanine aminotransferase (ALT), aspartate aminotransferase (AST), C-reactive protein (CRP), procalcitonin (PCT), activated partial thromboplastin time (APTT), and prothrombin time (PT), were collected 1 week before and 1 week after interventional therapy.

Treatment-related adverse events (TRAEs), including fever, pain, vomiting, hypertension, hypotension, chest distress, palpitation, hiccup, gastrointestinal bleeding, constipation, dysuria, thrombocytopenia, fatigue, and rash, were collected according to the common adverse events of TACE or HAIC reported in previous studies. Any adverse events occurring during the procedures or within 1 week postoperation were documented in detail.

### 2.3. The Ozone Therapy

Ozone therapy involves the preparation of a fresh mixture consisting of 5% ozone and 95% oxygen, referred to as medical ozone (MO). MO can be administered through various routes, including autologous blood therapy (autohemotherapy), rectal insufflation, or topical application. In this study, rectal ozone insufflation was used, which offers advantages such as being noninvasive and easy to perform. Ozone therapy is administered either before or after the patient undergoes interventional treatment. The procedure is as follows: first, a thin catheter is inserted approximately 5 cm into the patient's rectum via the anus; second, 150–300 mL of MO, at a concentration of 10–50 *μ*g/mL, is slowly infused. Each session lasts 30 min, and the procedure is performed once a day, with the patient's condition being closely monitored.

### 2.4. Statistical Analysis

Categorical variables were expressed as counts and percentages, while continuous variables were presented as means ± standard deviations or medians (interquartile range [IQR]). Group comparisons were performed using the Pearson's chi-square test or Fisher's exact test for categorical variables, and Student's *t* test or Mann–Whitney *U* test for continuous variables. Two-sided *p* values < 0.05 were considered statistically significant. All statistical analyses were conducted with R Statistical Software Version 4.5.1 and SPSS Statistics Package Version 29.0.

## 3. Results

### 3.1. Patient Characteristics

A total of 342 patients with HCC were enrolled in the analysis.  Table 1 presents the clinical characteristics of all patients. The majority of the patients were male (88.3%, 302/342), with a mean age of 56.2 ± 11.0 years. Most patients were classified as Child–Pugh Class A (83.3%, 259/311) and had evidence of liver cirrhosis (79.8%, 273/342). A total of 71.6% (245/342) of patients received TACE therapy, while 28.4% (97/342) received HAIC therapy. Of the total, 221 patients were enrolled in the ozone cohort and 121 patients in the control cohort. Most clinical characteristics were broadly balanced between the two cohorts (Table 1).

### 3.2. Comparison of TRAE Incidence

A total of 189 patients (55.3%) reported TRAEs of any grade, with no treatment-related deaths occurred. Among all adverse events, pain and fever had higher incidences, reported in 36.5% (125/342) and 24.9% (85/342) of patients, respectively. In the ozone cohort, 122 patients (55.2%) reported TRAEs of any grade, compared with 67 patients (55.4%) in the control cohort (*p* > 0.05). Regarding specific TRAEs, pain occurred in 35.7% (79/221) and fever in 26.2% (58/221) of the ozone cohort, while in the control cohort, pain was reported in 38.0% (46/121) and fever in 22.3% (27/121) (all *p* > 0.05). The overall occurrence of adverse events is summarized in Table 2. Notably, there were no significant differences between the two cohorts in terms of any grade of TRAEs.

### 3.3. Independent Risk Factors for TRAEs

Univariate and multivariate logistic regression analyses were conducted to identify risk factors associated with adverse events following interventional therapy (Table 3). The univariate analysis revealed that higher pretreatment levels of AFP and ALT/AST ratio were correlated with TRAEs in all 342 patients. Age, gender, type of interventional therapy, and ozone therapy did not show significant associations with TRAEs. In multivariate analysis, AFP (OR, 1.82; 95% CI, 1.13–2.94; *p* = 0.014) and ALT/AST ratio (OR, 2.02; 95% CI, 1.04–3.91; *p* = 0.037) emerged as significant independent risk predictors for the occurrence of TRAEs.

Moreover, independent predictive factors for specific TRAEs, including pain and fever, were also evaluated. Regarding pain, multivariate logistic regression analysis revealed that higher AFP levels (OR, 1.95; 95% CI, 1.21–3.17; *p* = 0.006) were associated with an increased risk of pain in patients with HCC (Table S1). For fever, male gender (OR, 2.49; 95% CI, 1.15–6.25; *p* = 0.032) was identified as an independent predictive factor for a higher risk of fever (Table S2).

### 3.4. Correlation Between Changes in Laboratory Parameters and Ozone Therapy

**Figure 2:**
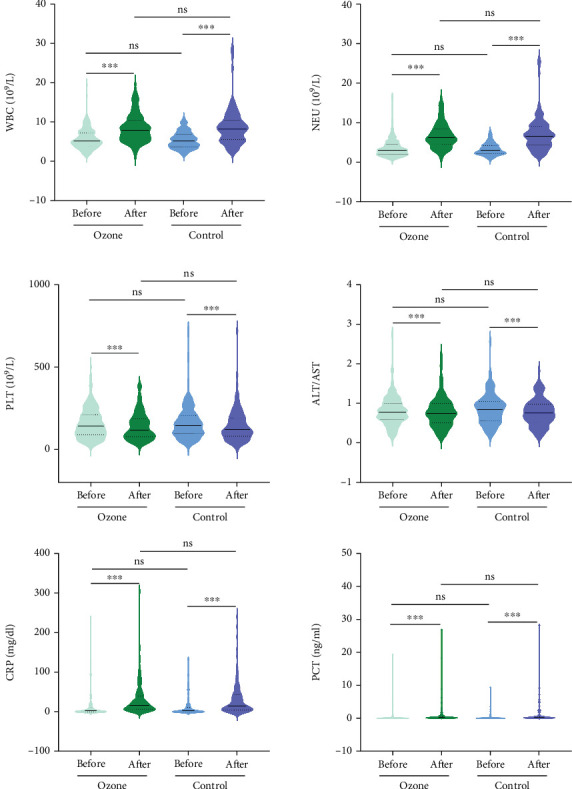
Laboratory parameters 1 week before and after interventional therapy in the ozone and control cohorts. WBC, white blood cell; ENU, neutrophil; PLT, platelet count; ALT, alanine aminotransferase; AST, aspartate aminotransferase; CRP, C-reactive protein; PCT, procalcitonin. ns: > 0.05; ∗∗∗: ≤ 0.001.

To assess changes in biomarkers related to ozone therapy, we analyzed laboratory parameters collected 1 week before and after interventional therapy. The results indicated similar levels of WBC, NEU, PLT, AST/ALT ratio, CRP, and PCT between the ozone and control cohorts before therapy. After interventional treatment, both cohorts showed significant increases in WBC, NEU, CRP, and PCT levels, while PLT and ALT/AST ratio exhibited a notable decrease. Furthermore, we compared postoperation levels of these clinical indicators between the ozone and control cohorts, and no significant differences were found (Figure 2).

## 4. Discussion

In this study, we compared the changes in the incidence of TRAEs and laboratory parameters between the ozone and control cohorts, exploring the effect of ozone therapy in patients with HCC undergoing interventional therapy. Our results indicated that the ozone therapy did not significantly reduce the incidence of adverse events or attenuate the increase in inflammatory markers in patients with HCC undergoing interventional therapy.

To the best of our knowledge, no study has previously assessed the impact of ozone therapy in patients with HCC undergoing interventional therapy. In our study, we firstly evaluate the effect of ozone therapy on TRAEs in these patients. The results suggest that the ozone cohort did not demonstrate better safety outcomes: the incidence of adverse events in patients receiving ozone therapy was similar to that in the control cohort (55.2% vs. 55.4%, *p* > 0.05). Additionally, there was no significant difference in the occurrence of any TRAEs between the two cohorts. This finding appears to be inconsistent with previous research. To our knowledge, ozone therapy is widely used in the management of musculoskeletal disorders and has been shown to effectively improve chronic inflammation and alleviate pain [10–12]. For instance, a prospective, open-label clinical study demonstrated that 540 patients with cervical and shoulder pain experienced a significant reduction in pain over time after receiving paravertebral muscle injections of an oxygen–ozone mixture [12]. Additionally, a case-series study revealed that the oxygen–ozone therapy through major autohemotherapy offers a promising opportunity for patients with breast cancer to reduce pain, fatigue, and musculoskeletal symptoms induced by antiaromatase treatment [13]. Unlike the ozone autohemotherapy and intramuscular injections used in the previous studies, the ozone therapy approach in this study involved rectal insufflation, which may account for the differences between our findings and those of the aforementioned research.

Another aspect worth exploring is whether the characteristics of patients with HCC influence the incidence of TRAEs associated with interventional therapy. To investigate this, we conducted univariate and multivariate logistic regression analyses, which revealed that higher levels of AFP and the ALT/AST ratio were significant independent risk factors for the occurrence of adverse events, while ozone therapy showed no association with TRAEs. These findings could provide valuable insights for managing patients with HCC and selecting the optimal therapy to minimize TRAEs. Moreover, independent predictive factors for specific TRAEs, including pain and fever, were also evaluated. Our analysis found that higher levels of AFP remain an independent risk factor for the occurrence of pain, while male gender was independently associated with an increased risk of fever. Although the correlation between male gender and fever remains unclear, this finding offers valuable insights for the practical prevention of fever in patients with HCC.

Additionally, we analyzed the correlation between changes in laboratory parameters and ozone treatment and found that ozone therapy did not slow the rise of inflammatory markers following interventional therapy. Despite similar pretreatment levels of WBC, NEU, PLT, ALT/AST ratio, CRP, and PCT in patients with HCC, no significant differences were observed in these biomarker levels posttreatment between the ozone and control cohorts. However, numerous studies have demonstrated that MO possesses anti-inflammatory effects. For instance, several studies showed the positive effects of ozone therapy as a complementary treatment in the recovery of patients with COVID-19, with ozone therapy significantly improving levels of D-dimer, lactate dehydrogenase (LDH), CRP, and interleukin-6 (IL-6) compared with standard therapy alone [14–16]. Furthermore, a study on severed finger replantation found that intervention with ozone autohemotherapy can significantly increase the number of WBC, alleviate postoperative pain, and enhance the survival rate of the reattached finger [17]. In the present study, compared with the control cohort, the ozone cohort exhibited slightly lower postoperative levels of WBC and NEU, though these differences were not statistically significant. A case-series observational study (involving six patients) demonstrated ozonated blood via major autohemotherapy reduced the extent of wound infections and decreased inflammatory biomarkers by more than 75% [18]. It is important to note that these studies primarily utilize ozone autohemotherapy, whereas our research employs rectal insufflation. The limited efficacy of ozone rectal insufflation in our study may be attributed to factors such as insufficient depth of catheter insertion into the rectum, a limited number of insufflation sessions, or a short follow-up period. These factors could help explain the discrepancies between our findings and those of previous studies.

MO can be administered via various routes, including autohemotherapy, rectal insufflation, or topical application. However, there is limited prior research on the rectal insufflation ozone therapy used in our study. In a controlled study investigating rectal insufflation ozone therapy for patients with multiple sclerosis, it was reported that the levels of proinflammatory cytokines, such as tumor necrosis factor-*α* (TNF-*α*) and interleukin-1*β* (IL-1*β*), were lower after ozone treatment compared with the control group [19]. A study also suggested that ozone therapy may help improve chronic renal tubular interstitial inflammation in rats; the results revealed that ozone therapy alleviated severe renal insufficiency and systemic electrolyte disorder, accompanied by a reduction in inflammation-related cytokines, including monocyte chemoattractant protein-1 (MCP-1), TNF-*α*, IL-1*β*, and IL-6 [20]. However, Kocaman's study showed there was no significant reduction in postoperative levels of AST, alkaline phosphatase (ALP), and direct bilirubin (DBil) in the ozone therapy group of rats compared with the control group [21]. Because of the absence of specific proinflammatory cytokines, such as IL-6 and TNF-*α*, in the routine clinical tests for patients with HCC undergoing interventional treatment at our hospital, our analysis was limited in its ability to fully explore ozone therapy's effects on inflammatory pathways. In this study, we focused on laboratory parameters including WBC, NEU, PLT, AST/ALT ratio, CRP, and PCT, while we did not explore the proinflammatory cytokines mentioned in previous studies. Therefore, the anti-inflammatory effects of rectal ozone insufflation therapy on patients with HCC undergoing interventional treatment remain to be further investigated.

To our knowledge, there is limited research on the application of ozone therapy in liver diseases. One study demonstrated that among patients with chronic hepatitis C undergoing ozone autohemotherapy, 57.5% and 60% of patients in the ozone group experienced a return to normal ALT and AST levels, respectively, compared with the control group [22]. This finding differs from our study, which may be attributed to variations in the ozone therapy method or differences in the type of liver disease among the patient populations. Additionally, a rat experimental study showed that ozone exposure has a protective effect on liver tissue, reducing inflammatory markers and alleviating oxidative stress [23], while another rat study indicated that ozone exposure led to significant chronic inflammation, fibrosis, and functional abnormalities in the liver [24]. Therefore, the potential of ozone therapy to alleviate liver inflammation still requires further investigation.

This study has several limitations. First, as a single-center retrospective cohort study, there may be recall bias from patients and documentation bias from clinicians or nurses responsible for recording patient information. Additionally, we were unable to fully retrieve data on the grade of TRAEs, which needs to be addressed in future studies. Second, the observational period in our study was relatively short, which may limit our ability to assess long-term TRAEs in patients with HCC treated with interventional therapies.

In conclusion, this is the first definitive analysis to assess the effect of ozone therapy in patients with HCC undergoing interventional treatment. Our results indicate that ozone therapy did not significantly reduce the incidence of adverse events or mitigate the increase in inflammatory markers. Additionally, we identified certain clinical characteristics in patients with HCC that may increase the occurrence of TRAEs, which could aid in decision-making and TRAE prevention in HCC treatment. Future studies should investigate the effect of ozone therapy on a larger cohort of patients with HCC receiving interventional treatment.

## Figures and Tables

**Table 1:** Clinical characteristics of all patients.

	**Total (** **n** = 342**)**	**Ozone cohort (** **n** = 221**)**	**Control cohort (** **n** = 121**)**	**p** ** values**
Age, years	56.2 ± 11.0	56.3 ± 11.2	56.2 ± 10.6	0.929
Male, *n* (%)	302 (88.3)	199 (90)	103 (85.1)	0.176
BMI, *n* (%)				0.005
≤ 25 kg/m^2^	265 (77.9)	162 (73.3)	103 (86.6)	
> 25 kg/m^2^	75 (22.1)	59 (26.7)	16 (13.4)	
Interventional therapy, *n* (%)				0.561
TACE	245 (71.6)	156 (70.6)	89 (73.6)	
HAIC	97 (28.4)	65 (29.4)	32 (26.4)	
BCLC stage, *n* (%)				0.636
A	79 (25.8)	53 (26.2)	26 (24.3)	
B	95 (31)	62 (31.2)	33 (30.8)	
C	131 (42.8)	83 (41.7)	48 (44.9)	
D	1 (0.3)	1 (0.5)	0 (0)	
Child–Pugh class, *n* (%)				0.106
A	259 (83.3)	169 (85.8)	90 (18.9)	
B	46 (14.8)	26 (13.2)	20 (17.5)	
C	6 (1.9)	2 (1)	4 (3.5)	
ECOG PS, *n* (%)				0.807
0	215 (68)	136 (68.3)	79 (67.5)	
1	85 (26.9)	54 (27.1)	31 (26.5)	
2	10 (3.2)	7 (3.5)	3 (2.6)	
3	10 (3.2)	2 (1)	4 (3.4)	
Cirrhosis, *n* (%)	273 (79.8)	178 (80.5)	95 (78.5)	0.655
AFP, *n* (%)				0.999
≤ 400 ng/mL	215 (65.5)	137 (65.6)	78 (65.5)	
> 400 ng/mL	113 (34.5)	72 (34.4)	41 (34.5)	
WBC, 10^9^/L	5.23 (3.96–7.20)	5.23 (4.00–7.33)	5.33 (3.80–6.90)	0.453
NEU, 10^9^/L	3.02 (2.12–4.30)	3.07 (2.06–4.50)	2.99 (2.20–4.21)	0.840
PLT, 10^9^/L	146 (92–211)	145 (91–213)	149 (98–208)	0.734
CRP, mg/dL	3.47 (1.21–12.74)	3.47 (1.26–13.10)	3.47 (1.16–12.02)	0.955
PCT, ng/mL	0.09 (0.04–0.19)	0.08 (0.04–0.18)	0.07 (0.04–0.20)	0.699
ALT, U/L	28 (20–41)	28 (20–41)	29 (19–44)	0.810
AST, U/L	35 (24–54)	35 (25–54)	36 (24–54)	0.638
APTT, S	27.2 (25.6–29.7)	27.2 (25.6–29.6)	27.3 (25.7–29.8)	0.473
PT, S	11.8 (11.2–12.8)	12.0 (11.4–13.2)	11.5 (11.2–12.3)	< 0.001

Abbreviations: AFP, alpha-fetoprotein; ALT, alanine aminotransferase; APTT, activated partial thromboplastin time; AST, aspartate aminotransferase; BCLC, Barcelona Clinic Liver Cancer; BMI, body mass index, weight (kg)/the square of height (m^2^); CRP, C-reactive protein; ECOG PS, Eastern Cooperative Oncology Group performance status; ENU, neutrophil; HAIC, hepatic artery infusion chemotherapy; PCT, procalcitonin; PLT, platelet count; PT, prothrombin time; TACE, transcatheter arterial chemoembolization; WBC, white blood cell.

**Table 2:** Incidence of treatment-related adverse events between ozone and control cohorts.

	**Total (** **n** = 342**)**	**Ozone cohort (** **n** = 221**)**	**Control cohort (** **n** = 121**)**	**p** ** values**
Total TRAEs	189 (55.3)	122 (55.2)	67 (55.4)	0.976
Pain	125 (36.5)	79 (35.7)	46 (38.0)	0.677
Fever	85 (24.9)	58 (26.2)	27 (22.3)	0.421
Vomiting	20 (0.06)	15 (0.07)	5 (0.04)	0.317
Hypertension	23 (0.07)	13 (0.06)	10 (0.08)	0.400
Hypotension	5 (0.01)	1 (0.005)	4 (0.03)	0.055
Chest distress	10 (0.03)	6 (0.03)	4 (0.03)	0.747
Dysuria	4 (0.01)	4 (0.02)	0 (0)	0.301
Palpitation	3 (0.01)	1 (0.005)	2 (0.02)	0.286
Gastrointestinal bleeding	3 (0.01)	2 (0.01)	1 (0.01)	1.000
Hiccup	2 (0.01)	1 (0.005)	1 (0.01)	1.000
Constipation	2 (0.01)	2 (0.01)	0 (0)	0.541
Thrombocytopenia	1 (0.003)	0 (0)	1 (0.01)	0.354
Fatigue	1 (0.003)	0 (0)	1 (0.01)	0.354
Rash	1 (0.003)	1 (0.005)	0 (0)	1.000

*Note:* Values are presented as *n* (%).

**Table 3:** Logistic regression analysis of total TRAE risk.

	**Univariate**		**Multivariate**	
	**OR (95% CI)**	**p** ** values**	**OR (95% CI)**	**p** ** values**
Age (> 56 vs. ≤ 56 years)	1.06 (0.69–1.62)	0.801		
Gender (male vs. female)	1.18 (0.66–2.11)	0.583		
BMI (> 25 vs. ≤ 25 kg/m^2^)	1.59 (0.94–2.72)	0.087		
Interventional therapy (TACE vs. HAIC)	1.20 (0.80–1.81)	0.380		
Ozone therapy (ozone vs. control)	0.99 (0.64–1.55)	0.976		
Cirrhosis (yes vs. no)	0.70 (0.40–1.18)	0.188		
BCLC stage (B + C + D vs. A)	1.66 (0.99–2.79)	0.054		
ECOG PS (≥ 1 vs. 0)	0.92 (0.58–1.49)	0.747		
AFP (> 400 vs. ≤ 400 ng/mL)	1.71 (1.07–2.74)	0.025	1.82 (1.13–2.96)	0.014
WBC	1.08 (0.98–1.19)	0.113		
NEU	1.07 (0.95–1.20)	0.298		
PLT	1.00 (1.00–1.00)	0.331		
CRP	1.00 (0.99–1.01)	0.875		
PCT	0.99 (0.96–1.03)	0.698		
ALT/AST	1.98 (1.07–3.76)	0.032	2.03 (1.06–4.01)	0.037
APTT	0.98 (0.92–1.04)	0.528		
PT	0.99 (0.96–1.03)	0.730		

Abbreviations: AFP, alpha-fetoprotein; ALT, alanine aminotransferase; APTT, activated partial thromboplastin time; AST, aspartate aminotransferase; BCLC, Barcelona Clinic Liver Cancer; BMI, body mass index, weight (kg)/the square of height (m^2^); CI, confidence interval; CRP, C-reactive protein; ECOG PS, Eastern Cooperative Oncology Group performance status; ENU, neutrophil; HAIC, hepatic artery infusion chemotherapy; OR, odds ratio; PCT, procalcitonin; PLT, platelet count; PT, prothrombin time; TACE, transcatheter arterial chemoembolization; TRAEs, treatment-related adverse events; WBC, white blood cell.

## Data Availability

The data generated during and/or analyzed during the current study are available from the corresponding authors on reasonable request.

## Nomenclature

ALT: alanine aminotransferase
AST: aspartate aminotransferase
CI: confidence interval
CRP: C-reactive protein
ENU: neutrophil
HAIC: hepatic artery infusion chemotherapy
HCC: hepatocellular carcinoma
MO: medical ozone
OR: odds ratio
PCT: procalcitonin
PLT: platelet count
TACE: transcatheter arterial chemoembolization
TRAEs: treatment-related adverse events
WBC: white blood cell

## Supporting Information

Supporting Information Additional supporting information can be found online in the Supporting Information section. Table S1: Logistic regression analysis of pain risk. Table S2: Logistic regression analysis of fever risk.
