# Supplementary material for: Effects of Ozone Therapy on Adverse Events and Inflammatory Markers in Patients With Hepatocellular Carcinoma Undergoing Interventional Therapy
Source: Gastroenterol Res Pract. 2025 Nov 12;2025:9953993. doi: 10.1155/grp/9953993 (PMC12629687; doi:10.1155/grp/9953993)
Supplement: Supporting Information — Additional supporting information can be found online in the Supporting Information section. Table S1: Logistic regression analysis of pain risk. Table S2: Logistic regression analysis of fever risk. [file 9953993.f1.docx]

Table S1: Logistic regression analysis of pain risk.

|  | Univariate | | Multivariate | |
| --- | --- | --- | --- | --- |
|  | OR (95%CI) | *p* values | OR (95%CI) | *p* values |
| Age (> 56 vs. ≤ 56 years) | 0.84 (0.54-1.31) | 0.445 |  |  |
| Gender (male vs. female) | 0.81 (0.45-1.48) | 0.486 |  |  |
| BMI (> 25 vs. ≤ 25 kg/m^2^) | 1.50 (0.89-2.53) | 0.126 |  |  |
| Interventional therapy (TACE vs. HAIC) | 1.46 (0.95-2.25) | 0.086 |  |  |
| Ozone therapy (ozone vs. control) | 0.91 (0.57-1.44) | 0.677 |  |  |
| Cirrhosis (yes vs. no) | 0.81 (0.47-1.40) | 0.437 |  |  |
| BCLC stage (B+C+D vs. A) | 1.51 (0.88-2.67) | 0.143 |  |  |
| ECOG PS (≥1 vs. 0) | 0.82 (0.50-1.35) | 0.442 |  |  |
| AFP (> 400 vs. ≤ 400 ng/mL) | 1.82 (1.14-2.91) | 0.013 | 1.95 (1.21-3.17) | 0.006 |
| WBC | 1.06 (0.97-1.16) | 0.226 |  |  |
| NEU | 1.07 (0.95-1.21) | 0.245 |  |  |
| PLT | 1.00 (1.00-1.00) | 0.444 |  |  |
| CRP | 1.00 (0.99-1.01) | 0.679 |  |  |
| PCT | 0.90 (0.61-0.99) | 0.401 |  |  |
| ALT/AST | 1.87 (1.02-3.51) | 0.046 | 1.68 (0.88-3.26) | 0.118 |
| APTT | 0.93 (0.87-1.00) | 0.042 | 0.95 (0.88-1.02) | 0.142 |
| PT | 1.00 (0.97-1.04) | 0.745 |  |  |

**Abbreviations**: TACE, transcatheter arterial chemoembolization; HAIC, hepatic artery infusion chemotherapy; BCLC, Barcelona Clinic Liver Cance; ECOG PS, Eastern Cooperative Oncology Group performance status; BMI, body mass index, weight (kg)/the square of height (m^2^); AFP, Alpha-Fetoprotein; PLT, platelet count; ALT, alanine aminotransferase; AST, aspartate aminotransferase; WBC, white blood cell; ENU, Neutrophil; CRP, C-reactive protein; PCT, procalcitonin; APTT, Activated partial thromboplastin time; PT, Prothrombin time; OR, odds ratio; CI, confidence interval.

Table S2: Logistic regression analysis of fever risk.

|  | Univariate | | Multivariate | |
| --- | --- | --- | --- | --- |
|  | OR (95%CI) | *p* values | OR (95%CI) | *p* values |
| Age (> 56 vs. ≤ 56 years) | 1.02 (0.62-1.66) | 0.951 |  |  |
| Gender (male vs. female) | 2.49 (1.15-6.25) | 0.032 | 2.49(1.15-6.25) | 0.032 |
| BMI (> 25 vs. ≤ 25 kg/m^2^) | 1.22 (0.68-2.15) | 0.497 |  |  |
| Interventional therapy (TACE vs. HAIC) | 0.90 (0.56-1.44) | 0.668 |  |  |
| Ozone therapy (ozone vs. control) | 1.24 (0.74-2.11) | 0.422 |  |  |
| Cirrhosis (yes vs. no) | 1.01 (0.56-1.91) | 0.963 |  |  |
| BCLC stage (B+C+D vs. A) | 1.53 (0.83-2.98) | 0.187 |  |  |
| ECOG PS (≥1 vs. 0) | 1.38 (0.79-2.38) | 0.253 |  |  |
| AFP (> 400 vs. ≤ 400 ng/mL) | 1.36 (0.81-2.27) | 0.240 |  |  |
| WBC | 1.05 (0.95-1.17) | 0.304 |  |  |
| NEU | 1.07 (0.94-1.22) | 0.269 |  |  |
| PLT | 1.00 (1.00-1.00) | 0.380 |  |  |
| CRP | 1.01 (1.00-1.02) | 0.085 |  |  |
| PCT | 1.00 (0.98-1.02) | 0.700 |  |  |
| ALT/AST | 1.30 (0.66-2.53) | 0.445 |  |  |
| APTT | 1.02 (0.96-1.10) | 0.492 |  |  |
| PT | 0.96 (0.81-1.02) | 0.513 |  |  |

**Abbreviations**: TACE, transcatheter arterial chemoembolization; HAIC, hepatic artery infusion chemotherapy; BCLC, Barcelona Clinic Liver Cance; ECOG PS, Eastern Cooperative Oncology Group performance status; BMI, body mass index, weight (kg)/the square of height (m^2^); AFP, Alpha-Fetoprotein; PLT, platelet count; ALT, alanine aminotransferase; AST, aspartate aminotransferase; WBC, white blood cell; ENU, Neutrophil; CRP, C-reactive protein; PCT, procalcitonin; APTT, Activated partial thromboplastin time; PT, Prothrombin time; OR, odds ratio; CI, confidence interval.
